# Ride-hailing services: Competition or complement to public transport to reduce accident rates. The case of Madrid

**DOI:** 10.3389/fpsyg.2022.951258

**Published:** 2022-07-27

**Authors:** María Flor, Armando Ortuño, Begoña Guirao

**Affiliations:** ^1^Department of Civil Engineering, University of Alicante, Alicante, Spain; ^2^University Institute of the Water and the Environmental Sciences, University of Alicante, Alicante, Spain; ^3^Department of Transport Engineering, Regional and Urban Planning, Universidad Politécnica de Madrid UPM, Madrid, Spain

**Keywords:** ride-hailing, road safety, injuries, traffic fatalities, public transport

## Abstract

**Introduction:**

The transport and mobility sector is experiencing profound transformations. These changes are mainly due to: environmental awareness, the increase in the population of large urban areas and the size of cities, the aging of the population and the emergence of relevant technological innovations that have changed consumption habits, such as electronic commerce or the sharing economy. The introduction of new services such as Uber or Cabify is transforming urban and metropolitan mobility, which has to adapt to this new scenario and the very concept of mobility.

**Objective:**

Thus, the purpose of this study was to evaluate whether ride-hailing platforms substitute or complement public transport to reduce accident rates, considering the two basic transport zones of Madrid: “The Central Almond” and the periphery.

**Methods:**

The data were collected from the 21 districts of Madrid for the period 2013–2019, and they were analyzed by a Random Effects Negative Binominal model.

**Results:**

The results obtained in this study suggest that since the arrival of Uber and Cabify to the municipality of Madrid the number of fatalities and serious injuries in traffic accidents has been reduced. Traffic accidents on weekends and holidays, with at least one serious injury or death, have also been reduced. However, the number of minor injuries has increased in the central districts of Madrid.

**Conclusion:**

Overall, what was found in this study supports the hypothesis that these services replace the urban buses. However, these services improve the supply to users with greater difficulties to access taxis or public transport, constituting an alternative mode of transport for high-risk drivers. Therefore, such findings may be quite useful for policy makers to better define regulatory policies for these services.

## Introduction

Mobility in cities has evolved in parallel with the development of large metropolitan areas, based on a central nucleus and an orbit of satellite towns which have numerous dependent relationships with the principal city. This phenomenon of dispersion, which is currently occurring in many other Spanish cities, together with the increase in the urban population, is possibly one of the factors that has most influenced the increase in metropolitan mobility and a greater use of private vehicles as opposed to other more efficient and sustainable solutions. In fact, the forecasts on a global level (International Transport Forum, 2017) indicate that the demand for urban passenger transport will grow intensely in the next few years (by 60–70% before 2050) and that this increase in demand will be more relevant in the case of motorized mobility (an increase of 94% between 2015 and 2050).

However, transport activity generates a series of negative impacts or externalities such as emissions of polluting and greenhouse gases, noise, accidents, congestion, etc. These factors negatively affect citizens' health and quality of life, the economy and the climate. The metropolitan areas, especially the urban centers, are hubs of activity and, mobility. Hence, the negative impacts associated with transport are concentrated in these areas.

Traffic accidents are highly relevant for sustainable mobility in urban environments if we take into account their high social costs. In addition, public policies designed to mitigate congestion can have unintended and usually unexpected indirect effects on road safety by modifying traffic conditions (Albalate and Fageda, 2019). Concerning the first consideration, the social cost of traffic accidents continues to be very high. According to the European Commission (2018) around 25,000 traffic fatalities occur each year in Europe. In addition, accidents also cause thousands of minor and serious injuries each year. It is calculated that for each death on European roads four permanently disabling injuries are sustained, such as damage to the brain or spinal cord, eight serious injuries and fifty minor injuries. In 2020, more than one million accidents occurred, with 1.4 million injuries and 25,651 fatalities. The external costs of traffic accidents were estimated at 1.7% of GDP in 2008 (Albalate and Fageda, 2019).

Within this context, technological platforms that place users and drivers in contact with one another in real-time have emerged. The model of these ride-hailing services is based on a technological application of on-demand transport, whereby an App acts as an intermediary to connect individuals who need to travel from one place to another with drivers (with a CDV license) willing to transport them. These applications are downloaded free of charge. They are easy to install and simple to use and the tariffs established by the businesses are lower than those of the traditional taxi. In Spain, the platforms available are Uber, Cabify and Mytaxi. These services constitute a good alternative to the use of private vehicles, which are parked for 95% of their useful life (Yaraghi and Ravi, 2017), representing a cost of between 5,000 and 7,000 euros per year when the depreciation of the vehicles is taken into account. The digital age has changed the way young people think. Smart phones and tablets have replaced the car as an icon of freedom. This is reflected in a survey carried out in Madrid, which reveals that six out of every ten young people between 18 and 25 years of age chose to purchase a mobile phone/tablet instead of a car (Cañigueral, 2014). This change is also visible in the way they choose the mode of transport. For example, with respect to how these platforms influence or coexist with the traditional economy (Pan and Qiu, 2018), after comparing the Uber platform with the traditional taxi services, Cramer and Krueger (2016) conclude that UberX had a higher capacity utilization rate than that of the taxis. Even in countries with a meager rate of digitization, Uber is one of the most widely used applications, showing a growing appreciation and perceived usefulness among applications potentially applicable to the traffic environment (Alonso et al., 2022). With regard to other forms of transport, there is limited knowledge of how these services have affected them, particularly the public transport system. On the one hand, these ride-hailing services can compete with the public transport system, providing another alternative mode of transport. In fact, the research reveals that the arrival of Uber in the United States is associated with a decrease in the use of the bus (Babar and Burtsch, 2017; Pan and Qiu, 2018) and light railway. However, it could be a complementary mode of transport for suburban trains (net increase of 3% in its use). Meanwhile, using a survey-based method, Clewlow and Mishra (2017) find that the people who use this type of ride-hailing platform are less likely to use public transport. On the other hand, Uber and Cabify can complement public transport as they connect the users with the transport centers, covering the “last mile”. Suburban trains usually provide a service to residents of the periphery of the cities distributed in a relatively wide geographical area (Vuchic, 2005). As a result, the last section of the journey (last mile) is not usually directly covered by the public transport operator and the users have to find a way to get to and return from the transport stop. This last mile of the journey can imply the use of a private vehicle, for example, driving one's own vehicle and parking it during the day (park and ride) (Noel, 1998); having a work colleague or classmate take you home (kiss and ride) (Schank, 2002) or hiring Uber or Cabify services.

Usually, the public transport services have the potential to complement the suburban rail services, helping the users to cover the last mile relatively comfortably and easily. On the other hand, the urban bus services usually operate so as to provide the widest possible coverage in residential areas, stopping in a relatively large number of places, every few blocks. In this way, the potential benefits of connecting the last mile are significantly reduced by the urban bus services (Babar and Burtch, 2020).

The changes perceived in the cities where these services operate have aroused great interest among researchers who have analized, among other aspects, how these platforms influence traffic congestion (Li et al., 2016), the local consumption of durable goods (Gong et al., 2017) or local business activity (Burtch et al., 2018). Most of these studies focus on cities in the United States, except those conducted in Chile, South Africa, Great Britain and Spain (Huang et al., 2019; Lagos et al., 2019; Kirk et al., 2020; Flor et al., 2022). Some of these studies observe a reduction in the cases of driving under the influence of alcohol (Meyer, 2016; Greenwood and Wattal, 2017; Peck, 2017; Dills and Mulholland, 2018; Morrison et al., 2018; Flor et al., 2021). This, could affirm that Uber and Cabify can constitute an alternative mode of transport used by high-risk drivers (such as drunk drivers) who run a higher risk of having a serious accident. However, if former public transit users are the predominant Uber customer rather than moderately risky drivers (e.g., such as those who are sleep deprived), the number of moderately risky drivers on the road would not decline. Therefore, the number of cars and even the number of moderate risky drivers could increase, which could lead to a rise in minor injuries (Kirk et al., 2020), particularly in a dense city like Madrid, where Uber use is greater. Therefore, this study analyses the evolution of the number of minor injuries, serious injuries and deaths in the districts of Madrid since the arrival of Uber and Cabify. If these services truly constitute an alternative mode of transport for high-risk drivers, we should observe a reduction in the number of serious injuries and deaths, with a lower impact on those districts with a higher presence of public transport. However, if these services are a substitute for public transport, the number of minor injuries could remain the same or even increase in relation to the figures observed before the arrival of these platforms.

### Objective of the study

The main objective of this research was to study whether ride-hailing services, such as Uber and Cabify, substitute or complement public transport to reduce accident rates. Moreover, a comparison was made between the two differentiated transportation zones in Madrid: the “Central Almond” and the periphery.

## Materials and methods

### Madrid case study

Although Madrid is one of the cities in Spain with the highest supply of public transport per inhabitant, the level of pollution still exceeds the legal limits of air quality according to Directive 2008/50/EC (City Council of Madrid, 2016). Therefore, in recent decades, the city council has designed pollution protocols to apply during high air pollution episodes and has promoted low emissions parking policies (Guirao et al., 2021). In this respect, Madrid is the Spanish city with the highest incidence of free-floating, whereby the user hires the vehicle from their smartphone, for short periods of time (usually for minutes or hours) and picks it up and leaves it in the street. These are very attractive services for clients who wish to use a vehicle occasionally, as they do not have the need to purchase their own. These include mainly car-sharing services, followed by moto-sharing, bike-sharing, and scooter-sharing. The actors offering these new mobility services are principally the companies providing the services (Sharenow -formerly Car2go-, Emov, Zity, Wible, Whisilife, Muving, eCooltra, Ioscoot, Movo, Acciona, Eskay, Voi, Scoot, Bolt, Circ, Jump Uber, etc.) (Ministry of Transportation Mobility Urban Agenda, 2020). Furthermore, November 30 2018, the Residential Priority Area (APR) or Low Emission Zone (ZBE) was established, which restricts access to the most polluting vehicles in the Central District of Madrid (1) with the objective of complying with the nitrogen dioxide limit values established by the basic community and state regulations on air quality: Law 34/2007, of November 15, 2007, on air quality and atmospheric protection (Law, 2007) and Royal Decree 102/2011, of January 28, 2011, on the improvement of air quality (Royal Decree, 2011; Madrid City Council web portal, n.d.).

**Figure 1:**
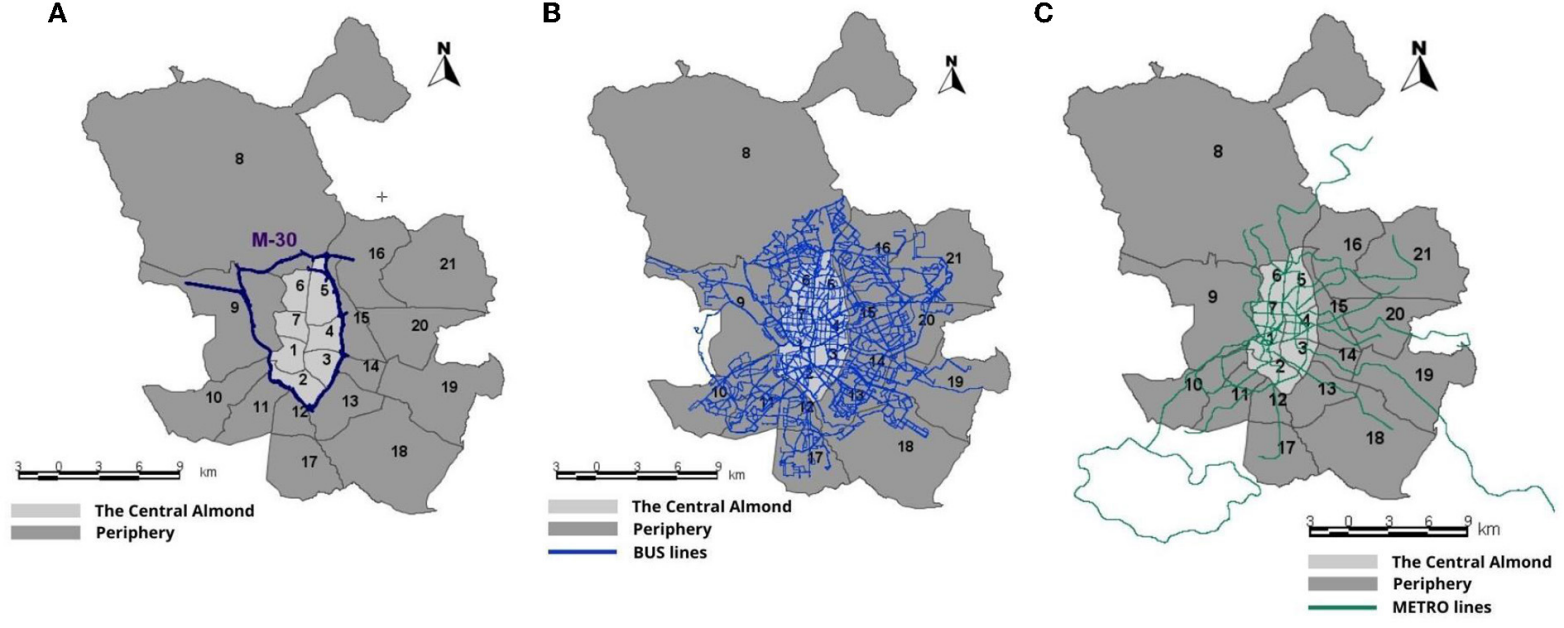
**(A)** Transport zones, **(B)** BUS lines, and **(C)** METRO lines. Source: Own elaboration. 1-Center; 2-Arganzuela; 3-Retiro; 4-Salamanca; 5-Chamartín; 6-Tetuán; 7-Chamberí; 8-Fuencarral-El Pardo; 9-Moncloa-Aravaca; 10-Latina; 11-Carabanchel; 12-Usera; 13-Puente de Vallecas; 14-Moratalaz; 15-Ciudad Lineal; 16-Hortaleza; 17-Villaverde; 18-Villa de Vallecas; 19-Vicálvaro; 20-San Blas-Canillejas; 21-Barajas.

For the basic division of the territory, the municipality of Madrid has a typical concentric radial structure based on its central nucleus called the “Central Almond” and which practically coincides with the urban area within the ring motorway M-30, traditionally made up of the seven districts of: Centro (1), Arganzuela (2), Retiro (3), Salamanca (4), Chamartín (5), Tetuán (6) and Chamberí (7), although the City Council of Madrid (8) also includes, as an eighth district Moncloa-Aravaca (9). The districts outside of this perimeter road constitute the “Madrid Periphery” (Madrid Regional Transport Consortium (CRTM), 2018) (Figure 1A).

These peripheral districts are urban development areas with hardly any connection with each other, mainly because the transport network is highly focused on center-periphery movements and practically ignores movements between peripheries (Figures 1B,C).

This is reflected in the data referring to modal distribution, by basic transport zones: more than half of the journeys starting or ending in the “Central Almond” are made on foot. The rest of the journeys are made on public transport (73% of which are motorized), with a very limited use of the car. Public transport is mostly used for radial journeys between the “Central Almond” and the periphery (two-thirds are motorized) as a result of the excellent radial supply of all public modes of transport. The regulation of the SER (Regulated Parking Service in the “Central Almond” has also decisively influenced the reduction in the use of cars. On the other hand, for journeys made wholly in the periphery, cars are the most used motorized modes of transport (56%). Furthermore, it is also where most car trips are made (1.1 million per day) which gives rise to congestion in the different ring motorways (M-30, M-40, M-45) of the city (City Council of Madrid, 2019).

Madrid also has a good database of road safety data to study traffic accidents. The quality of the available accident database largely determines the success and focus of any research on road safety. Compared with other national databases, the Spanish database is sufficiently consolidated (Casado et al., 2019a,b). The data for injuries and deaths used in this research have been downloaded from the open data portal of the City Council of Madrid (n.d.a). The downloaded files register the accidents causing injuries or damage to municipal assets, classified by district for a period of seven years (2013–2019).

The accidents are categorized in the data according to the severity of the injuries: “Serious” (anyone injured in a traffic accident and whose condition requires hospitalization for more than 24 h); “Minor” (anyone injured in a traffic accident to whom the definition of serious injury does not apply) and “Death” (anyone who, as a result of a traffic accident, dies on the spot or within 30 days following the accident) (Directorate-General for Traffic of Spain, 2016).

It is important to remember that there are other factors that can influence the severity of the accident. These factors can be classified into three groups: human, road and vehicle.

The human factor (driver or pedestrian) has a major role intriggering of accidents, as the vast majority of accidents involve human error at some point. For this reason, there is a large body of literature that has examined major intrinsic and extrinsic factors associated with the increased risk of fatal road crashes (Ghandour et al., 2020). From this bibliography it can be extracted that human factors represented the most common risk factors involved in road traffic fatalities and are present in 3 out of every 5 of the accidents produced (Petridou and Moustaki, 2000; Moradkhani et al., 2014; Razzaghi et al., 2019). Drivers' characteristics such as age, gender, safety measures adopted or risk-taking behavior, also influence the severity of crash outcomes (Duncan et al., 1999; Krull et al., 2000; Bédard et al., 2002; Rolison et al., 2018). Other factors that contribute to the severity of the accident are alcohol consumption and the time of the accident (O'Donnell and Connor, 1996; Duncan et al., 1999; Krull et al., 2000; Zajac and Ivan, 2002; Keall et al., 2004; Yau and Yau, 2004; El Tayeb et al., 2015). For example, in accidents that occurred from 1:00 to 5:00 h the risk of death is higher than from 6:00 to 11:00 h (Valent et al., 2002). Excessive speed, failure to use a seatbelt, single-vehicle, wet surfaces and road lighting conditions can also increase the risk of traffic fatalities (Krull et al., 2000; Al-Ghamdi, 2002; Altwaijri et al., 2011; Pakgohar et al., 2011).

As previous literature shows, the presence of alcohol plays an important role in the severity of the accident. According to World Health Organization data, some 55,000 young people between 15 and 29 years of age die annually in Europe as a result of alcohol consumption, mainly in traffic accidents (Del Río, 2002). In this sense, Spain occupies one of the highest places in terms of alcohol consumption rates and problems derived from its consumption. An analysis of the data provided by the National Institute of Toxicology regarding the presence of alcohol in fatal traffic accidents shows the presence of alcohol in 50.1% of the drivers killed in traffic accidents, with a blood alcohol level above 0.8 g/l in 32% of the cases (Luque, 2000).

In Section Introduction of this paper, it has been mentioned that Uber and Cabify may constitute an alternative mode of transportation used by high-risk drivers (such as drunk drivers) who are at an increased risk of a severe accident. However, if the former users of public transport are the principal clients of Uber, the number of moderate-risk drivers (for example, those who lack sleep) on the roads will not decrease. Fatigue and sleepiness are two human factors that negatively impact driving ability because they increase distractions, decrease the ability to concentrate and increase reaction times. Therefore, if Uber and Cabify replace public transportation, the number of cars and even the number of moderate-risk drivers could increase, which could lead to an increase in minor injuries.

The database consulted for this analysis only provides information about the presence of alcohol in traffic accidents in the year 2019. Therefore, we decided to also study the number of accidents (with at least one serious injury or death) at weekends (from Friday at 00:00 to Sunday at 23:59) and public holidays.This is because, together with night driving, they represent a risk for young people compared with the rest of the population (Directorate-General for Traffic of Spain, 2020), due to the increase in the consumption of alcohol at these times (Anowar et al., 2013). In fact, the majority of drivers dying under the age of 34 who tested positive for alcohol consumption lost their lives at the weekend and public holidays (Ministry of Justice, 2020). Even work-related traffic accidents with serious injuries are more frequent at weekends and public holidays (Useche et al., 2020). In 2018, Madrid recorded a total of 1,225 accidents with positive alcholemia - and refusal to test - of which 64.4% occurred between Friday and Sunday. The day of the week with the highest number of fatalities was Friday (Madrid Municipal Police, 2018).

In view of the above, the variables of analysis selected are:

- The annual number of minor injuries in traffic accidents,
- The annual number of serious injuries in traffic accidents,
- The annual number of accidents (with at least one serious injury or death) at the weekend and public holidays.

**Figure 2:**
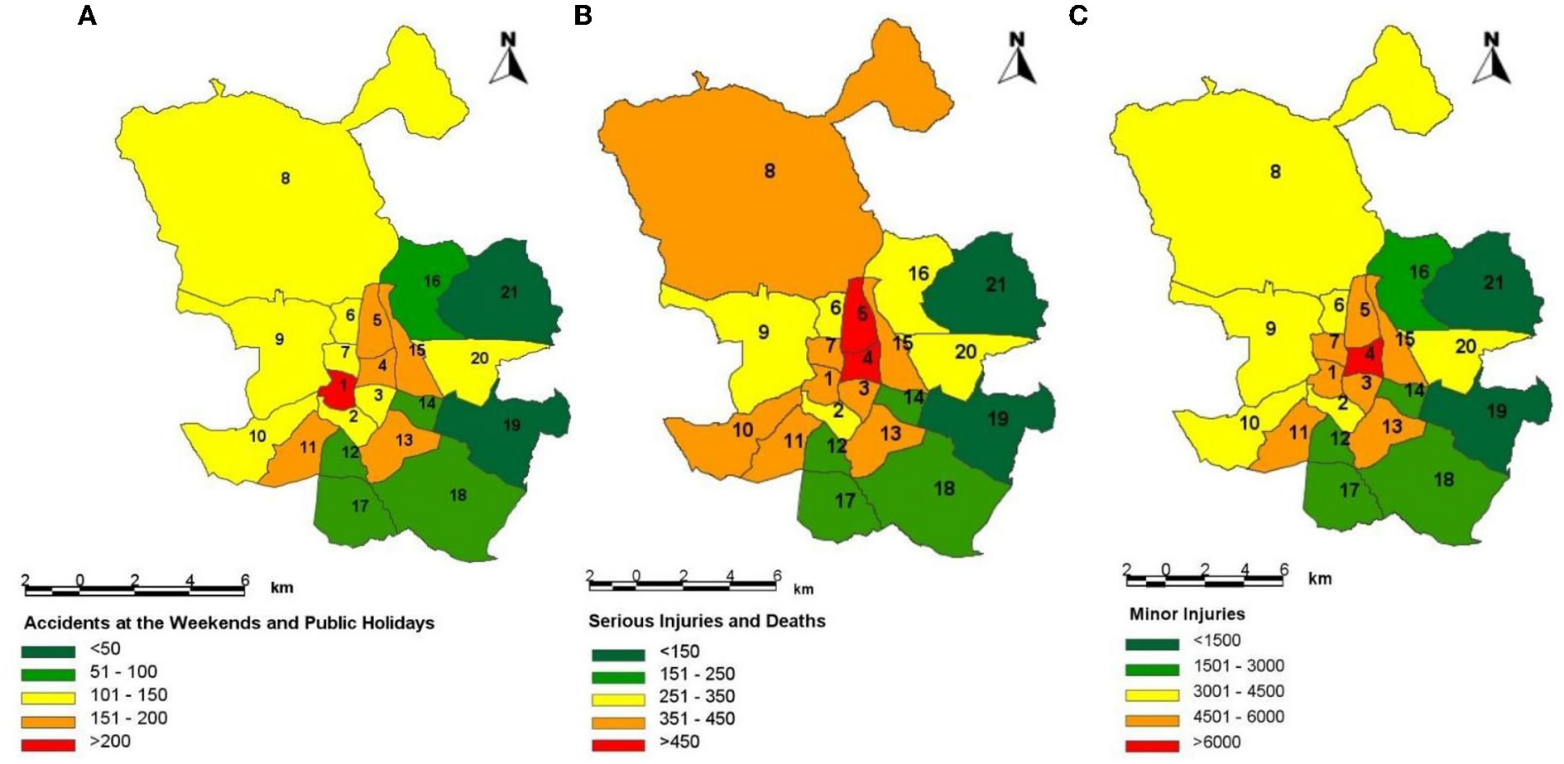
**(A)** Number of accidents at weekends and public holidays; **(B)** number of serious injuries and deaths; and **(C)** number of minor injuries by district in Madrid in the period 2013–2019. Source: Own elaboration. 1-Center; 2-Arganzuela; 3-Retiro; 4-Salamanca; 5-Chamartín; 6-Tetuán; 7-Chamberí; 8-Fuencarral-El Pardo; 9-Moncloa-Aravaca; 10-Latina; 11-Carabanchel; 12-Usera; 13-Puente de Vallecas; 14-Moratalaz; 15-Ciudad Lineal; 16-Hortaleza; 17-Villaverde; 18-Villa de Vallecas; 19-Vicálvaro; 20-San Blas-Canillejas; 21-Barajas.

**Figure 3:**
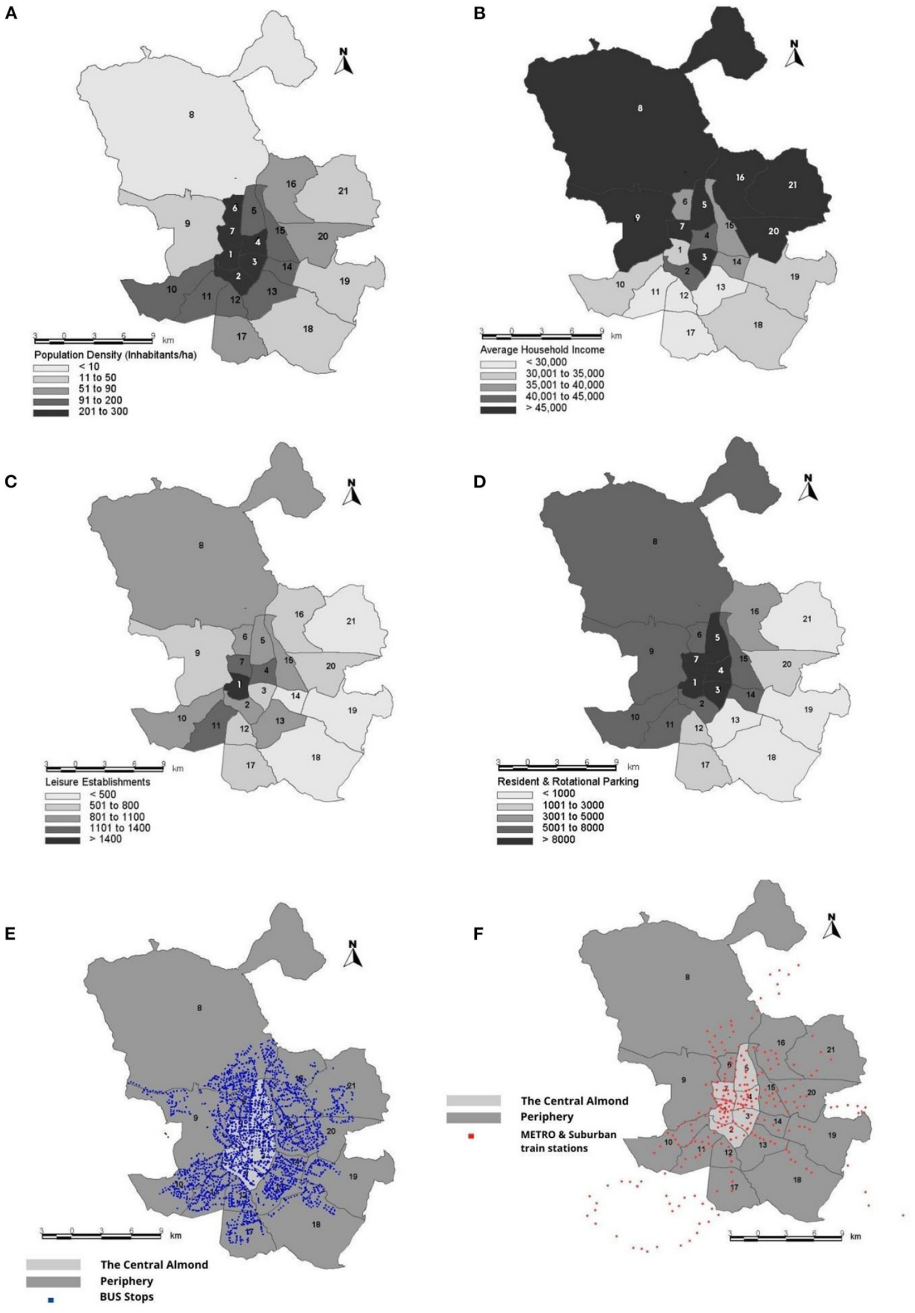
Spatial distribution of the independent variables analyzed: **(A)** Average population density in the districts of Madrid analyzed in the period 2013–2019; **(B)** Average household income in the districts of Madrid analyzed in the period 2013–2019; **(C)** Average number of leisure establishments in the districts of Madrid analyzed in the period 2013–2019; **(D)** Average number of parking spaces for residents and rotational spaces in the districts of Madrid analyzed in the period 2013–2019; **(E)** Location of the bus stops in the districts of Madrid; **(F)** Location of metro stations in the districts of Madrid. 1-Center; 2-Arganzuela; 3-Retiro; 4-Salamanca; 5-Chamartín; 6-Tetuán; 7-Chamberí; 8-Fuencarral-El Pardo; 9-Moncloa-Aravaca; 10-Latina; 11-Carabanchel; 12-Usera; 13-Puente de Vallecas; 14-Moratalaz; 15-Ciudad Lineal; 16-Hortaleza; 17-Villaverde; 18-Villa de Vallecas; 19-Vicálvaro; 20-San Blas-Canillejas; 21-Barajas.

Figure 2 shows the three dependent variables of this analysis by district of Madrid for the period 2013–2019. The number of accidents at weekends and public holidays with at least one serious injury or death is higher in the district of the Center (1). As shown in Figure 2A, this could be because this district concentrates the highest number of leisure establishments. Furthermore, and as could be expected, the urban center or “Central Almond” (districts 1, 3, 4, 5 and 7) has a higher concentration of injuries and deaths compared to the periphery as it is a focal point of activity (Figures 2B, 3C).

The ride-hailing variable is a binary indicator that varies over time and indicates the availability of Uber and Cabify in Madrid. In order to determine the year in which Uber and Cabify services started operating in Madrid, the evolution of the revenues of Uber System Spain S.L and Maxi Mobility Spain S.L (Cabify), and the evolution CDV licenses (chauffeur-driven vehicle) in the Community of Madrid were consulted (Flor et al., 2021).

Furthermore, some of the most used variables in the literature on road safety were also gathered and processed (Wedagama et al., 2006; Wier et al., 2009; Ukkusuri et al., 2012) and classified into five groups: (I) socioeconomic variables, (II) land uses (III) public transport and costs, (IV) transport policy variables and (V) climatology. The majority of studies conducted on accidents and/or victims include a variable of exposure which enables the results obtained to be compared unanalyzed. The most appropriate unit for this end is vehicles-km, but due to the lack of data, an approximate measure was used, namely population (Aparicio et al., 2008).

The results obtained in previous studies show a direct relationship between traffic accidents and the economic level of a country, with fewer accidents occurring in times of recession and increasing numbers in periods of economic growth (Aparicio et al., 2008). Therefore, in this study the socioeconomic status of each district of Madrid was also considered (Figure 3B).This variable was calculated based on the average income per household and district, following the methodology used in the report conducted by the “Methodology for calculating the territorial vulnerability index of neighborhoods and districts of Madrid and the vulnerability ranking” (2018) (Territorial Coordination Public-Social Cooperation Area, 2018; Flor et al., 2021). Figure 3A shows each district's average population density analyzed from 2013–2019. A higher level of population density could generate a higher traffic density and, as a result, increase the number of traffic accidents. Scheiner and Holz-Rau (2011) and Casares et al. (2019) find that urban residents have a higher risk of accidents with injuries than the population of other areas.

Land use and street configuration can influence the incidence of traffic accidents. Even certain commercial uses are associated with an increased risk of accidents (Dumbaugh and Rae, 2009). Therefore, one of the variables considered in this work is the number of leisure establishments available in each district (Figure 3C).

The fuel price is an important consideration in the choice of the mode of transport (Wang and Skinner, 1984; Lane, 2010; Iseki and Ali, 2014), not only for public transport options but also for ride-hailing services. If the price of petrol increases and the ride-hailing services do not raise the price of the service, the drivers' profit margins decrease and the drivers' interest in providing the service also reduces. This leads to a lower number of cars in service and, therefore, an increase in the waiting times of the clients. On the other hand, given that the dynamic price algorithms used by the ride-hailing services respond to imbalances in supply and demand, a reduction in the number of operators will increase the prices of the service. Hence, it would be expected that the value proposition of these services could be reduced with the increase in the price of petrol, while that of the public transport options could remain relatively stable in the short term (Babar and Burtch, 2020).

In order to estimate the presence of public transport in each of the district analized, the potential population of each bus stop and metro and suburban train station has been taken into account (Figures 3E,F). To do this, the potential demand in the surrounding area of each stop and station has been calculated (Gallego et al., 2014). In theory, a larger public transport infrastructure should be associated with a better and more efficient public transport which should affect the overall relative cost of the transport in favor of the mass modes. Recent studies indicate that users' perception of the quality and safety of public transport may influence their choice of transport mode (Alonso et al., 2020, 2021). Furthermore, all of the public transport options are theoretically safer alternatives than private vehicles, so the number of accidents could decrease. However, a greater use of public transport will reduce the number of vehicles on urban roads, which could lead to a higher average speed with the resulting negative effect in terms of road safety (Albalate and Fageda, 2019).

As mentioned in Section Introduction of this study, the transport policies developed in the municipality of Madrid (directly associated with the regulation of mobility) could have indirect effects on traffic accidents in the districts in which they are implemented. The stricter parking regulations are considered the second best option and an alternative to road pricing. For this reason, the category of parking regulation appears in most of the mobility plans analyzed in Spanish cities (Mozos-Blanco et al., 2018). In addition, one of the main factors affecting travelers' preference for the passenger car is the availability of parking space (Tyrinopoulos and Antoniou, 2013). This study includes a variable referring to the regulation of the parking spaces in the urban nucleus. This variable is determined by the number of resident spaces, which are areas that are usually more advantageous for the residents (low price or free) but less so for visitors and the number of short-term or rotational parking spaces (Albalate and Fageda, 2019) (Figure 3D). Furthermore, it is important to take into account the Residential Priority Area (APR) or Low Emission Zone (ZBE), which restricts access to the most contaminating vehicles in the Center District (1), and therefore, reduces the number of vehicles therein. Hence, a binary indicator has been included which identifies the years in which the restrictions were imposed in the Center District (0 without traffic restrictions and 1 with traffic restrictions).

As an additional explanatory variable, we have included the number of rainy daysin order to take into account traffic conditions. Although worse conditions should lead to a greater exposure to risk, Peltzman's compensatory behavior effect may arise (Peltzman, 1975). This behavioral effect could lead to less travel in wet conditions and more cautious driving. Thus offsetting the negative effects of adverse weather conditions (Albalate and Fageda, 2019). The data for this variable have been obtained from the State Meteorology Agency (AEMET).

However, there are other very important variables that could not be taken into account because they are not normally included in the accident databases. Some of these variables are: driver behavior and perceived aggressive behavior. In fact, behaviors that appear in the definition of aggressiveness (such as shouting, swearing, rude gestures, overtaking or crashing) are perceived as less aggressive than violations of the Highway Code (driving under the influence of alcohol, running red lights, speeding). This situation corresponds to a double quagmire for traffic and road safety research and intervention, since the data point to a huge number of accidents caused by these dangerous behaviors that are nevertheless perceived as less aggressive from a descriptive point of view (Alonso et al., 2019).

In Table 1, the variables considered in this analysis are defined.

**Table 1:** Variables source: own research.

**Variables**	**Definition**	**Units**	**Data Source**
Exposure			
Population	Number of inhabitants in each district analyzed	Inhab.	Data bank: Population by district and neighborhood (City Council of Madrid, n.d.b)
Primary variable			
Ride-hailing Services	Binary indicator 1: Uber or Cabify presence 0: neither Uber nor Cabify		
Socioeconomic			
Population density	Demographic variable that measures the number of people who live in each district, considering the population and area.	Inhab./ha	Statistical Yearbook Chapter I: Territory and Environment (City Council of Madrid, 2004a)
socioeconomic status	Indicator calculated based on the average income per household and district	%	Urban Audit (City Council of Madrid, n.d.c) and (National Institute of Statistics, 2020) (INE)
Land use leisure establishments	Variable that measures the number of establishments dedicated to the catering, leisure, and entertainment activities per hectare	Establishments/ha	Data bank. Economy: census of business premises and activities (City Council of Madrid, n.d.d)
Public transport and cost			
Bus stop	Variable that measures the potential demand of each stop, considering the population density (inhab./ha) and the density of stops (Bus Stop/ha).	Inhab./Bus St	Madrid Regional Transport Consortium (CRTM)
METRO & Suburban train stations	Variable that measures the potential demand of each station, considering the population density (inhab./ha) and the density of stations (Stations/ha).	Inhab./Stations	
Diesel cost	Average Diesel Cost	€/liter	(Ministry for the Ecological Transition, n.d)
Petrol cost	Average Petrol Cost	€/liter	
Transport policies			
Resident & rotational parking	Number of resident and rotational parking spaces in each district		Statistical Yearbook Chapter VII: Traffic and Transportation (City Council of Madrid, 2004b)
Low emission zone (LEZ)	Year and district dummy variables: 1, traffic restrictions in the Downtown District 0, no traffic restrictions in the Downtown District		
Weather			
Rain	Number of rainy days per year in Madrid		State Meteorology Agency (AEMET).

It should be noted that some explanatory variables are metric and continuous. Therefore, a logarithmic transformation should be applied to optimize them to eliminate problems such as heteroscedasticity or obtain variance-stabilizing variables' transformations in the statistical model (Casares et al., 2019). In this study, a logarithmic transformation has been applied “Log(X)”, where X is the value of each independent variable in each district. It has been applied to all of the explanatory variables except the fictitious variables (ride-hailing and Low Emission Zone), socioeconomic status, leisure establishments and price of fuel.

Table 2 provides descriptive statistics of the analyzed variables.

**Table 2:** Descriptive statistics.

	**Obs**.	**Maximum**	**Minimum**	**Standard deviation**	**Mean**
Dependent variables					
Minor injuries	147	1,065	130	215.431	564.014
Serious injuries and deaths	147	80	2	16.642	45.326
Accident at the weekend and Public holidays	147	36	1	8.628	17.939
Independent variables					
Exposure					
Population	147	261.118	45.721	54,451.08	153,421.7
Primary variable					
Ride-hailing	147	1	0	0.496	0.571
Socioeconomic					
Log population density	147	2.48	0.990	0.472	1.968
Socioeconomic status	147	7.465	7.090	0.105	7.326
Land use					
Leisure establishments	147	6.288	0.325	1.324	1.048
Public transport and cost					
Log bus stop	147	2.992	2.520	0.118	2.829
Log metro & suburban train station	147	4.491	3.657	0.218	4.095
Diesel cost	147	1.385	1.061	1.107	1.221
Petrol cost	147	1.483	1.216	0.086	1.345
Transport policies					
Log residential & rotational parking	147	4.237	2.324	0.488	3.544
Low emission zone	147	1	0	0.824	0.006
Weather					
Log rain	147	2.053	1.778	0.097	1.947

*Source: own research*.

### Statistical methods

The principal objective of this study is to assess the influence of ride-hailing services on the number of injuries and deaths and the number of accidents occurring at weekends and public holidays, considering the two basic transport zones (“Central Almond” and periphery). The analysis was conducted on a macroscopic scale, using the districts of Madrid as spatial units of reference. The district level was selected to use the data available in the official databases, which did not require a large amount of post-processing operations. The lowest level of detail found in these data was at the district level.

The Negative Binomial (NB) distribution is the regression model most used to explain the behavior and evolution of the frequency of different types of traffic accidents, including studies on the macroscopic level (Ziakopoulos and Yannis, 2020). The NB model is a distribution derived from the Poisson Gamma distribution (Hilbe, 2011) and has been used in this analysis because it can operate with over-dispersed data (this occurs if the variance is higher than the average). This characteristic cannot be found in a Poisson regression model, as the average and variance are identical. Given that overdispersion was found in the number of minor injuries, serious injuries and deaths and the number of accidents at weekends and public holidays in the overall data gathered by district, the NB model is appropriate for constructing the statistical models toanalize. The Probability Density Function (PDF) of the NB distribution is as follows (Hosseinpour et al., 2014; Gálvez et al., 2022):


$$\begin{array}{l} P\left(Y=y_{i}\right)=\frac{\Gamma \left(y_{i}+\alpha^{-1}\right)}{\Gamma \left(\alpha^{-1}\right)y_{i}!}{\left(\frac{\alpha {\cdot \mu }_{i}}{1+\alpha \cdot \mu_{i}}\right)}^{y_{i}}{\left(\frac{1}{1+\alpha \cdot \mu_{i}}\right)}^{\alpha^{-1}} \end{array}$$

P(Y = y_i_) is the probability that Y gives rise to y_i_, μ_i_ is the predicted number of accidents, injuries and deaths, y_i_ is the number of accidents, injuries and deaths in district i, and α is the dispersion parameter.

### Heterogeneity in panel data

Panel data, also called longitudinal data, are defined as any data set with repeated observations over time (Arellano, 2003). In other words, in contrast to the cross-sectional data, which are compiled at a single point in time, the longitudinal ones contain statistical units (N) that are measured more than once in time (T) (Pignataro, 2018).

As mentioned above, for this study, information has been collected in each of the districts of Madrid (*N* = 21), over 7 years (T = 7) -before and after the entry of Uber and Cabify-, obtaining a sample of NxT observations. Therefore, the collected information is presented in panel data.

The main objective of panel data models is to capture unobservable heterogeneity, ignored in traditional regression models, and which can somehow affect the estimation of the effects of the independent variables on the dependent variable (Perazzi and Merli, 2014).

In the case of traffic accidents, it is common for observations from the same group to be correlated in terms of space and/or time, due to unobserved factors that may exist in the same groups. One way to account for this correlation (or heterogeneity) as well unobserved heterogeneity is to apply a random-effect NB model. The random-effects negative binomial (RENB) model assumes that the within-segment heterogeneity is uncorrelated with the explanatory variables. If this assumption does not hold, a fixed-effect (FE) specification should be used (Hosseinpour et al., 2014). The selection between FE and RE models is based on a Hausman test, which determines which model is more appropriate (Hausman et al., 1984). If the test is insignificant, the RE model is preferred over the FE model, implying that there is no correlation between independent variables and random effects (Kweon and Kockelman, 2005).

Numerous studies have applied random effects models to the analysis of traffic accidents (Kumara et al., 2003; Huang and Chin, 2010; Quddus, 2013). For example, the RENB model was used to analyze the variables influencing traffic accidents at signalized intersections in Singapore (Chin and Quddus, 2003). Shankar et al. (1998) after their analysis in the State of Washington, concluded that from a predictive point of view, RENB offers greater advantages in terms of model transferability and updating. Kweon and Kockelman (2005) selected, the random effects negative binomial model among several alternative panel models to analyze the effects by modifying speed limits on 143 state highways in Washington State.

The expected number of casualties and deaths can be calculated using the following equation:


$$\begin{array}{l} \mu_{i}=\exp\left(\beta X_{it}+u_{i}+\varepsilon_{it}\right) \end{array}$$

where μ_it_ represents the expected number of casualties and deaths in the i^th^ district in year t, β is the vector of regression parameters, X_it_ is the vector of explanatory variables, u_i_ represents the random effect for the i^th^ location, ε_it_ is the vector of residual errors, and exp(u_i_) is gamma distributed with mean 1 and variance α_i_, where α_i_ is the parameter of overdispersion in the negative binominal model. The RENB model allows the overdispersion parameter to vary randomly from district to district, such that 1/(1 + α_i_) follows a Beta (r, s) distribution (Hausman et al., 1984; Shankar et al., 1998; Naznin et al., 2016; Flor et al., 2021).

For all of the above reasons, and after performing the goodness-of-fit tests described in section Heterogeneity in panel data of this paper, the model selected for this study is the random effects negative binomial (RENB).

The equations of the RENB model used in this study are described below:


$$\begin{array}{l} \;Minor\;Injures=Population^{\beta_{1}}\\ \;\cdot \;\exp(\beta_{2}\cdot \;{\left(Ride-hailing\right)}_{it}+\beta_{3}.Log{\left(Population\;Density\right)}_{it}\\ \;+\beta_{4}\;\cdot \;{\left(Socioeconomic\;Status\right)}_{it}+\beta_{5}\;\cdot \;{\left(Leisure\;Establishments\right)}_{it}+\beta_{6}\cdot Log{\left(Bus\;Stop\right)}_{it}\\ \;+\beta_{7}\cdot Log{\left(Metro\;\&Suburban\;train\;stations\right)}_{it}+\beta_{8}\cdot \;{\left(Diesel\;Cost\right)}_{it}\;+\beta_{9}\cdot \;{\left(Gasoline\;Cost\right)}_{it}\;\\ \;+\beta_{10}\cdot Log{\left(Resident\;\&\;Rotational\;parking\right)}_{it}+\beta_{11}\cdot \;{\left(Low\;Emission\;Zone\right)}_{it}\\ \;+\beta_{12}\cdot Log{\left(Rain\right)}_{it}+u_{i}+\varepsilon_{it})\\ \;Serious\;Injures\;and\;Deaths=Population^{\beta_{1}}\\ \;\cdot \;\exp(\beta_{2}\cdot \;{\left(Ride-hailing\right)}_{it}+\beta_{3}.Log{\left(Population\;Density\right)}_{it}\\ \;+\beta_{4}\cdot \;{\left(Socioeconomic\;Status\right)}_{it}+\beta_{5}\cdot \;{\left(Leisure\;Establishments\right)}_{it}+\beta_{6}\cdot Log{\left(Bus\;Stop\right)}_{it}\\ \;+\beta_{7}\cdot Log{\left(Metro\;\&\;Surburban\;train\;stations\right)}_{it}+\beta_{8}\cdot \;{\left(Diesel\;Cost\right)}_{it}+\beta_{9}\cdot \;{\left(Gasoline\;Cost\right)}_{it}\\ \;+\;\beta_{10}\cdot Log{\left(Resident\;\&Rotational\;parking\right)}_{it}+\beta_{11}\cdot {\left(Low\;Emission\;Zone\right)}_{it}\\ \;+\beta_{12}\cdot Log{\left(Rain\right)}_{it}+u_{i}+\varepsilon_{it})\\ Acc.weekend\;and\;Public\;Holidays=Population^{\beta_{1}}\\ \;\cdot \;\exp(\beta_{2}\cdot \;{\left(Ride-hailing\right)}_{it}+\beta_{3}.Log{\left(Population\;Density\right)}_{it}\\ \;+\beta_{4}\cdot {\left(Socioeconomic\;Status\right)}_{it}+\beta_{5}\cdot {\left(Leisure\;Establishments\right)}_{it}+\beta_{6}\cdot Log{\left(Bus\;Stop\right)}_{it}\\ \;+\beta_{7}\cdot Log{\left(Metro\;Suburban\;train\;stations\right)}_{it}+\beta_{8}\cdot {\left(Diesel\;Cost\right)}_{it}+\beta_{9}\cdot {\left(Gasoline\;Cost\right)}_{it}\\ \;+\;\beta_{10}\cdot Log{\left(Resident\text{ \& }Rotational\;parking\right)}_{it}+\beta_{11}\cdot {\left(Low\;Emission\;Zone\right)}_{it}\\ \;+\beta_{12}\cdot Log{\left(Rain\right)}_{it}+u_{i}+\varepsilon_{it}) \end{array}$$

### Goodness-of-fit of the model

The maximum likelihood estimation method was used to measure the goodness of fit of the model, so instead of the classic coefficient of determination, the McFadden (1973) pseudo-squared value was used, based on the following equation:


$$\begin{array}{l} R^{2}=1-\frac{LL\left(\beta \right)}{LL\left(C\right)} \end{array}$$

LL(β) is the log-likelihood value of the full model, and LL(C) is the log-likelihood value of the constant-only model (Naznin et al., 2016).

Miaou et al. (1996) proposed another measure, $R_{\alpha }^{2}$, to determine the extent to which the variance of the data is captured by the model relative to a fundamental model without variables (Shahla et al., 2009). This measure takes the dispersion parameter NB and is estimated as follows:


$$\begin{array}{l} R_{\alpha }^{2}=1-\frac{\alpha }{1+\alpha_{max}} \end{array}$$

α is the estimated overdispersion parameter for the selected model and α_max_ is the estimated overdispersion parameter for the fundamental model containing the only constant term (Hilbe, 2011).

In addition, selecting the most suitable model, the Bayesian information criterion (BIC) has been used based partly on the likelihood function and closely related to the Akaike information criterion (AIC).

A model with the lowest AIC and BIC values is preferred.

## Results

### Goodness-of-fit model and selection

Table 3 compares the results of the random effects negative binomial (RENB) and negative binomial (NB) models.

**Table 3:** Results of the fitted models.

	**Serious injuries and deaths**		**Minor injuries**		**Accident at the weekend and Public Holidays**	
**Models**	**NB**	**RENB**	**NB**	**RENB**	**NB**	**RENB**
Log-Likelihood at convergence	−625.2949	−625.2949	−666.6002	−966.6002	−501,857	−501,857
Log–likelihood with constant only	−542.4282	−534.0994	−852.7459	−769.2218	−436,359	−431.1889
McFadden pseudo R^2^	0.133	0.146	0.118	0.204	0.131	0.141
AIC	1110.856	1096.199	1731.492	1566.444	898.7182	890.3777
BIC	1149.732	1138.065	1770.368	1608.31	937.5939	932.2438
Dispersion parameter, α(95%CI)	0.0283 (0.0183–0.0437)		0.0223 (0.0174–0.0286)		0.0196 (0.008–0.0492)	
${\text{R}}_{\text{a}}^{2}$	0.9753		0.98		0.983	
/ln_r		5.247 (10.46)		4.329 (12.58)		6.365 (5.87)
/ln_s		4.979 (9.29)		4.707 (12.99)		4.291 (7.47)
LR test vs. pooled: chibar2(01)		12.81		178.88		8.92
Prob≥chibar2		0.000		0.000		0.001
Hausman Test: chi2(8) Prob > chi2		14.82 0.0723 > 0.05		10.21 0.5120 > 0.05		5.27 0.9174 > 0.05

*Source: Own research*.

First, it should be noted that in the three analized models, the dispersion parameter estimated from the NB model (α) was significantly different from zero, suggesting that the negative binomial model structure was more appropriate than the Poisson structure. Moreover, the NB model formulation can explain most of the variations in collision data because the value ${\text{R}}_{\text{α}}^{2}$ is more than 0.7 in all three models (Miaou et al., 1996; Shahla et al., 2009; Naznin et al., 2016).

However, the RENB model improves the overall fit (R^2^ = 0.146 Serious injuries and deaths; R^2^ = 0.204 minor injuries; R^2^ = 0.141 Accident at the weekend and public holidays) in comparison with the NB model (R^2^ = 0.133 Serious injuries and deaths; R^2^ = 0,118 Minor injuries; R^2^ = 0.131 Accident at the weekend and public holidays). The result of the likelihood ratio test, compared with the combined test indicates that the panel estimate is significant in comparison with the pooled estimate.

The results also show that both AIC and BIC give RENB an advantage over the NB model by obtaining lower values after testing.

In addition, the Hausman specification test is not significant. Therefore we fail to reject the null hypothesis of equal estimates, so the most efficient estimator is that of random effects.

### Main analysis

Table 4 presents the results of the three dependent variables studied. The “Ride-hailing” variable is significant (*p* < 0.001) in the three models. Moreover, in models 1 and 3, it can be observed that the emergence of these services is associated with a significant decrease in the number of deaths and serious injuries, of around 28.5% and a reduction in the number of accidents at weekends and public holidays with at least one death or serious injury, of around 25.7%.

**Table 4:** Results of the estimated Model (z-statistics in parentheses).

	**Dependent variables**		
**Independent variables**	**Model 1 Serious injuries and deaths**	**Model 2** **Minor injuries**	**Model 3 Accident at the weekend and Public Holidays**
Primary variable			
Ride-hailing	−0.285*** (−6.68)	0.0621*** (3.42)	−0.257*** (−4.75)
Socioeconomic			
Log population density	0.386** (3.26)	0.369** (3.11)	0.345* (2.50)
Socioeconomic status	−0.645 (−1.11)	−0.744 (−1.58)	−0.819 (−1.23)
Land use			
Leisure establishments	0.0626 (1.34)	0.0789* (2.40)	0.133* (2.50)
Public Transport and Costs			
Log BUS stop	– 1.166* (– 2.43)	– 0.974* (– 2.16)	– 1.251* (– 2.24)
Log METRO-suburban train station	−0.531^+^ (−1.67)	−0.310 (−1.23)	−0.176 (−0.48)
Diesel cost	−5.851*** (−5.82)	0.186 (0.59)	−5.179*** (−4.07)
Gasoline cost	6.508*** (4.92)	−0.178 (−0.43)	5.738*** (3.44)
Transport Policies			
Log Residential & Rotational Parking	– 0.0366 (– 0.35)	0.00880 (0.11)	– 0.0007 (– 0.01)
Low Emission Zone	−0.870** (−2.86)	−0.0805 (−1.25)	−0.733* (−2.27)
Weather			
Log Rain	−0.0405 (−0.17)	−0.0941 (−1.21)	0.02 (0.07)
Exposure			
Ln (Population)	1	1	1
Log likelihood	– 534.0994	– 796,222	−431,189
Wald chi2	208.18	114.40	130.73
Prob>chibar	0.000	0.000	0.000
/ln_r	5.247*** (10.46)	4.329*** (12.58)	6.365*** (5.87)
/ln_s	4.979*** (9.29)	4.707*** (12.99)	4.291*** (7.47)
LR test vs. pooled: chibar2 (01)	12.81	178.88	8.92
Prob ≥ chibar2	0.000	0.000	0.001

+p <0.1; *p <0.05; **p <0.01; ***p <0.001. Source: own research.

However, the number of minor injuries (model 2) increased by around 6%.

Additionally, the principal objective of this analysis was to evaluate whether ride-hailing platforms substitute or complement public transport to reduce accident rates, considering the two basic transport zones of Madrid: “The Central Almond” and the periphery. For this, it was necessary to create a new interaction variable between the ride-hailing variable and each group analyzed.

The results of Table 5 show that the coefficients of the interaction term of Uber/Cabify entry and the transport zone analyszed are significant in the three models. In this respect, the number of serious injuries and deaths (model 1) has fallen both in the “Central Almond” and in the periphery. However, the impact of these platforms has been greater in the peripheral districts (with the number of serious injuries and deaths falling by 54.9% as opposed to 30.5% in the “Almond”). Also, the number of accidents (with at least one serious injury or death) has decreased in both zones (Model 3), with the impact being more remarkable in the peripheral zones (50% as opposed to 3%).

**Table 5:** Results of the estimated Model (z–statistics in parentheses).

	**Dependent variables**		
**Independent variables**	**Model 1 Serious injuries and deaths**	**Model 2** **Minor injuries**	**Model 3 Accident at the weekend and Public Holidays**
Socioeconomic			
Log population density	0.288*** (2.38)	0.246* (1.96)	0.259^+^ (1.77)
Socioeconomic status	−0.110 (−0.16)	−0.435 (−0.86)	−0.282 (−0.35)
land use			
Leisure establishments	0.0511 (1.07)	0.0816* (2.15)	0.121* (2.17)
Public transport and costs			
Log BUS stop	– 1.678** (−3.06)	−1.492** (−3.10)	0.0663 (0.17)
Log METRO-suburban train station	−0,251 (−0.78)	0.0891 (0.30)	−0.176 (−0.48)
Diesel cost	−5.848*** (−5.83)	0.212 (0.68)	−5.174*** (−4.07)
Petrol cost	6.517*** (4.95)	−0.198 (−0.48)	5.749*** (3.45)
Transport policies			
Log residential and rotational parking	−0.0295 (– 0.27)	0.0422 (0.53)	0.0668 (0.49)
Low emission zone	−0.836** (−2.75)	−0.0706 (−1.09)	−0.699* (−2.16)
Weather			
Log Rain	−0.0493 (−0.20)	−0.0927 (−1.19)	0.0132 (0.04)
Interaction			
Uber/Cabify entry*Central Almond	−0.305*** (−4.91)	0.0571* (2.02)	−0.0278*** (−3.57)
Uber/Cabify entry*Periphery	−0.549*** (−3.63)	−0.246^+^ (−1.65)	−0.497** (−2.65)
Exposure			
Ln (Population)	1	1	1
Log likelihood	−532.27079	−767.019	−430.147
Wald chi2	223.64	133.33	138.61
Prob>chibar	0.000	0.000	0.000
/ln_r	5.426*** (10.41)	4.508*** (13.24)	6.512*** (5.77)
/ln_s	5.139*** (9.15)	4.880*** (13.60)	4.391*** (7.32)
LR test vs. pooled: chibar2 (01)	10.40	165.50	7.71
Prob ≥ chibar2	0.001	0.000	0.003

Source: Own research. +p <0.1; *p <0.05; **p <0.01; ***p <0.001.

Minor injuries (Model 2) have also decreased by 25% in the peripheral districts of the city [although the coefficient is weakly significant (*p* < 0.1)]. These districts are more dependent on private vehicles because public transportation infrastructure is scarcer in these areas. But it is possible that residents in these peripheral areas of the city have been attracted to these services, for their flexibility and the low cost of the service. This would have reduced the number of private vehicles and consequently, the number of moderate risk drivers (more likely to suffer a minor accident).

However, the number of minor injuries has increased by around 5.7% in the “Almond”. It should be emphasized that in this zone, 73% of the journeys are made by public transport [33]. This increase in the number of minor injuries would reinforce the assumption that Uber and Cabify will replace public transport, particularly the urban buses.

## Discussion

The results reveal that since the entry of ride-hailing services in Madrid, the number of fatalities and serious injuries in traffic accidents has been reduced. These results are compatible with those obtained by Kirk et al. (2020) where, after analyzing traffic accidents in cities in Great Britain, they found a reduction in the number of serious injuries. However, the introduction of UberCAR in the Dominican Republic was associated with an increase of 0.03 in the level of monthly car occupant deaths per 100,000 inhabitants in Santo Domingo, and with an increase of 0.20 in Santiago. These variations in the results might reflect differences in specific city features, including characteristics of the vehicle fleet and public transportation systems (Nazif-Munoz et al., 2022).

Traffic accidents with at least one serious injury or death on weekends and holidays, have also been reduced. These results could confirm the hypothesis that these services have become an alternative mode of transport for high-risk drivers who have consumed alcohol or young people who are returning home after a night of partying -as service users are mainly between 18 and 25 years of age (Huynh et al., 2020). The individual dynamic price system, which determines the price of the service through the use of technology, enables the Uber drivers to offer a specific supply whenever they want (increasing or decreasing their rates). For example, in the US, on special nights such as Halloween or New Year's Eve, Uber prices can increase as much as 3 fold. This increase in prices encourages drivers to work at times of high demand, resulting in an increase in supply, and generating confidence among users who know that they will be able to rely on a service that guarantees their return home after a night out, even if they live on the outskirts of the city (Meyer, 2016). These results are also supported by the study carried out by Qian et al. (2020) where they identified that the weekend evening rush hour attracts twice as many drivers as the weekday morning rush hour.

A comparison of Madrid's two different transport zones shows that the reduction in accident rates has had a greater impact in the peripheral zones. This would reinforce the hypothesis that this type of service acts as an alternative mode of transport for people who may have consumed alcohol, as it guarantees a safe mode of transport to get home when the public transport service is limited (nights and weekends). Furthermore, it is possible that the entry of these new services has improved the supply to demand segments which formerly had greater difficulties to accessing taxis, such as low-income families or residents in the periphery of the cities. When the number of vehicles is lower, they tend to be concentrated in the more profitable zones of the market (central areas) leaving the areas relatively further away without a service. In this regard, the study by Qian et al. (2020) found that Uber services avoid midtown Manhattan on weekdays during peak hours. One possible reason for this behavior could be the high traffic congestion in the area, which could suggest that drivers during those hours prefer peripheral areas.

However, Uber may be a substitute for cabs and other forms of public transportation, but not for moderately risky driving (i.e., the type of drivers who suffer “minor” injury accidents). Consequently, if transportation *via* Uber is a substitute for public transportation, then the number of cars and even the number of risky drivers (e.g., those with sleep deprivation) may even increase, resulting in the increase in the observed number of minor injuries (Kirk et al., 2020).

On the other hand, it is important to point out that this reduction in the accident rate in certain areas could also be related to the 2012–2020 Road Safety Plan approved by the Madrid City Council. The main objective of this Plan was to prevent accidents and protect people's life, health and physical integrity using public roads and spaces. To this end, since 2012 the Madrid City Council has developed annual road safety education campaigns in schools, and universities, for people over 65 years of age, minor offenders, etc. Other parallel measures to these prevention campaigns were the installation of more speed radars on the streets of Madrid and an increased number of police controls (Environment, Safety and Mobility Government Area and Security and Emergencies Area, 2012).

### Limitations of the study and the future lines of research

As with most academic studies that have analyzed the relationship between traffic accidents and the entry of these ride-hailing services, this study also has limitations. First, there is no individual-level information available on trips, drivers and users of these services, and therefore, it has not been possible to determine whether drivers who have consumed alcohol or other high-risk drivers use these services. We also do not know the condition of the drivers of these services (tiredness, hours of driving, etc.) (Kirk et al., 2020; Flor et al., 2021) nor adverse problems present in the psychosocial environment of professional driving and these play an important role in accidents (Useche et al., 2019). In terms of public transport, nor do we know the number of users on a district level in order to verify whether the number of bus and metro users has reduced since the arrival of Uber and confirm the replacement of certain modes of transport with Uber or Cabify.

Future studies could address these points using complementary data sources and analyzed the demand for public transport in accordance with the ride-hailing supply. Hoffman et al. (2016), for example, studied the effect of the closure of Metro stations and the demand for ride-hailing services close to them. Other modes of transport could also be incorporated into the analysis, such as taxis or bicycle sharing (Babar and Burtsch, 2017).

Therefore, this study constitutes an initial effort in this field. We expect subsequent research to extend this line of study to improve the collective understanding of the impacts of from the new ride-hailing services.

## Conclusions

The results obtained suggest that the number of minor injuries has increased in the central zone of Madrid (“Central Almond”) which could indicate that Uber and Cabify are replacing public transport, particularly the urban buses. However, the impact of these services has been greater in the peripheral districts of Madrid, where a reduction in the number of injuries and deaths in traffic accidents has been observed since the entry of these services.

In general, in people depend more on private vehicles for their journeys in these areas because public transport is inefficient. However, the results obtained indicate that after the entry of these new more flexible and economic operators, the residents of these more distant districts could have been attracted by these services, preferring them to private vehicles. Furthermore, the door-to-door service they offer removes the need of the users find a parking space at the destination, saving time which benefits their health, reduces congestion and adapts mobility to the needs of the citizens. Therefore, these services will have contributed to improving connectivity in these peripheral areas, broadening the people's employment opportunities and fostering economic growth. Moreover, these services could improve the integrated urban transport system without the need to invest in more resources as these new activities would serve the mobility needs of modes of transport with a higher potential to generate congestion (private vehicles) and would increase the reach of the urban integrated transport network (last mile) (AFI, 2017). For example, an Uber vehicle could cover the last mile of a bus journey. After analyzing aggregate Uber trip data in the Miami metropolitan area, it was found that most riders used public transportation to the closest station of their destination and then used Uber as a connection to the location of interest (Roy et al., 2020). The user could request the Uber service while on the bus and the vehicle would be waiting at the stop to cover the last mile of the journey. In this way, the efficiency of collective transport service is combined with the flexibility of a private vehicle (Feigon and Murphy, 2016).

These findings may be quite useful for policy makers to better define regulatory policies for these services, since if these types of services really reduce traffic fatalities in Madrid, promoting their use could be advantageous for the city.

Finally, we should not attribute this change in the accident rate in certain areas solely to the entry of Uber and Cabify since, as discussed throughout this paper, there are other factors such as the Road Safety campaigns carried out in Madrid since 2012, the installation of radars and control devices, etc., which may have also contributed to the reduction in traffic accidents.

## Data availability statement

Publicly available datasets were analyzed in this study. This data can be found here: The datasets presented in this study can be found in online repositories. The names of the repository/repositories can be found below: Traffic accidents in the City of Madrid: https://datos.madrid.es/portal/site/egob/menuitem.c05c1f754a 33a9fbe4b2e4b284f1a5a0/?vgnextoid=7c2843010d9c3610 VgnVCM2000001f4a900aRCRD&amp;vgnextchannel=374 512b9ace9f310VgnVCM100000171f5a0aRCRD&amp;vgnex tfmt=default Population by district and neighborhood. Available online at: http://www-2.munimadrid.es/TSE6/control/seleccionDatosBarrio Leisure Establishments: http://www-2.munimadrid.es/CSE6/control/seleccionDatos?numSerie=4020101010 Household income: https://www.madrid.es/portales/munimadrid/es/Inicio/El- Ayuntamiento/Estadistica/Areas-de-informacion-estadistica/ Economia/Renta/Urban-Audit/?vgnextfmt=default&amp;vgnex toid=6d40393c7ee41710VgnVCM2000001f4a900aRCRD &amp;vgnextchannel=ef863636b44b4210VgnVCM2000000c 205a0Arcrd, https://www.ine.es/jaxiT3/Datos.htm?t=31097 BUS Stops and METRO & Suburban train stations: https://data-crtm.opendata.arcgis.com/ Resident & rotational parking: https://www.madrid.es/portales/munimadrid/es/Inicio/El -Ayuntamiento/Estadistica/Anuario-Estadistico-Municipaldes 2004-/?vgnextfmt=default&amp;vgnextoid=7e1e63af4fe4631 de-0VgnVCM2000000c205a0aRCRD&amp;vgnextchannel= 8156e39873674210VgnVCM1000000b 205a0aRCRD.

## Author contributions

AO and BG conceived the idea and reviewed the whole manuscript. MF designed the study, collected the data, performed the statistical analysis, and wrote the original draft. AO supervised the study during all phases. All authors contributed to the article and approved the submitted version.

## Funding

María Flor García is currently developing her doctoral thesis on sharing economy and mobility and received an FPU grant from the University of Alicante: UAFPU2018–028.

## Conflict of interest

The authors declare that the research was conducted in the absence of any commercial or financial relationships that could be construed as a potential conflict of interest.

## Publisher's note

All claims expressed in this article are solely those of the authors and do not necessarily represent those of their affiliated organizations, or those of the publisher, the editors and the reviewers. Any product that may be evaluated in this article, or claim that may be made by its manufacturer, is not guaranteed or endorsed by the publisher.
